# A systematic review of the use and effectiveness of social media in child health

**DOI:** 10.1186/1471-2431-14-138

**Published:** 2014-06-02

**Authors:** Michele P Hamm, Jocelyn Shulhan, Gillian Williams, Andrea Milne, Shannon D Scott, Lisa Hartling

**Affiliations:** 1 Alberta Research Centre for Health Evidence, Department of Pediatrics, Faculty of Medicine and Dentistry, University of Alberta, Edmonton, Alberta, Canada; 2 Faculty of Nursing, University of Alberta, Edmonton, Alberta, Country; 3 4-488B Edmonton Clinic Health Academy, 11405 – 87 Avenue, Edmonton, Alberta T6G 1C9, Canada

**Keywords:** Social media, Pediatrics, Systematic review

## Abstract

**Background:**

Social media use is highly prevalent among children, youth, and their caregivers, and its use in healthcare is being explored. The objective of this study was to conduct a systematic review to determine: 1) for what purposes social media is being used in child health and its effectiveness; and 2) the attributes of social media tools that may explain how they are or are not effective.

**Methods:**

We searched Medline, CENTRAL, ERIC, PubMed, CINAHL, Academic Search Complete, Alt Health Watch, Health Source, Communication and Mass Media Complete, Web of Knowledge, and Proquest Dissertation and Theses Database from 2000–2013. We included primary research that evaluated the use of a social media tool, and targeted children, youth, or their families or caregivers. Quality assessment was conducted on all included analytic studies using tools specific to different quantitative designs.

**Results:**

We identified 25 studies relevant to child health. The majority targeted adolescents (64%), evaluated social media for health promotion (52%), and used discussion forums (68%). Most often, social media was included as a component of a complex intervention (64%). Due to heterogeneity in conditions, tools, and outcomes, results were not pooled across studies. Attributes of social media perceived to be effective included its use as a distraction in younger children, and its ability to facilitate communication between peers among adolescents. While most authors presented positive conclusions about the social media tool being studied (80%), there is little high quality evidence of improved outcomes to support this claim.

**Conclusions:**

This comprehensive review demonstrates that social media is being used for a variety of conditions and purposes in child health. The findings provide a foundation from which clinicians and researchers can build in the future by identifying tools that have been developed, describing how they have been used, and isolating components that have been effective.

## Background

The popularity of social media has changed the way healthcare providers and consumers access and use information, providing new avenues for interaction and care. This advancement of technology has created an environment in which individuals have the opportunity to participate and collaborate in the sharing of information, and may be particularly relevant for children and youth. In a 2013 report on adolescents’ use of social media and mobile technology, researchers from the Pew Internet and American Life Project found that 95% of teens surveyed used the Internet, a figure that has remained constant in the United States since 2006 [1]. Additionally, 73% of teens have a cell phone, of which almost half are smartphones [1]. In 2012, Lenhart et al. [2] reported that when teens possess a smartphone, more than 90% use it to connect with social networking sites. Even without such a highly connected mobile device, 77% of teens still logged into social networking sites, and overall, almost 50% sent daily text messages to their friends [2]. Furthermore, many teens employ multifaceted methods to communicate with their peers, including the Internet, instant messaging, and social networking sites [3].

Considering the extensive degree of connectivity exhibited by today’s youth, it may be worthwhile for healthcare providers to find ways to engage with teens in forums in which they are already comfortable interacting. Some success has been achieved in the use of mobile technology (i.e., instant messaging, text messages) for increasing medication adherence and appointment attendance, and it has been noted that many adolescents are using the Internet to find health information, especially on sensitive topics (e.g., sexual health, drug use) [4]. Given this context, the use of social media tools may be an effective strategy in developing healthcare interventions for children and youth.

There is clear interest in how new technologies can be used to improve patient outcomes, including in children and youth, therefore we conducted a systematic review to answer two key questions: 1) for what purposes are social media being used in the healthcare context for children, youth, and their families, and are they effective for these purposes; and 2) what are the attributes of the social media tools used in this population that may explain how they are or are not effective.

## Methods

This systematic review was based on a scoping review that we conducted to determine how social media is being used in healthcare [5]. Child health emerged as an area for further study, therefore the scoping review was used as a foundation, and the search was updated with a focus on children, youth, and their families.

### Search strategy

A research librarian searched 11 databases in January 2012: Medline, CENTRAL, ERIC, PubMed, CINAHL, Academic Search Complete, Alt Health Watch, Health Source, Communication and Mass Media Complete, Web of Knowledge, and Proquest Dissertation and Theses Database [5]. Dates were restricted to 2000 or later, corresponding to the advent of Web 2.0. No language or study design restrictions were applied. The search was updated in May 2013. The search strategy for Medline is provided in the Additional file 1.

### Study selection

Two reviewers independently screened titles and abstracts of studies for eligibility. The full text of studies assessed as ‘relevant’ or ‘unclear’ was then independently evaluated by two reviewers using a standard form. Discrepancies were resolved by consensus or adjudication by a third party.

Studies were included if they reported primary research, with analytic quantitative designs used to answer whether social media is effective for use in child health, and descriptive and qualitative designs used to provide context to attributes that may contribute to the effectiveness or lack of effectiveness of the tools being studied. Further, studies were included if they focused on children, youth, or their families or non-professional caregivers, and examined the use of a social media tool. Social media was defined according to Kaplan and Haenlein’s classification scheme [6], including: collaborative projects, blogs or microblogs, content communities, social networking sites, and virtual worlds. We excluded studies that examined mobile health (e.g., tracking or medical reference apps), one-way transmission of content (e.g., podcasts), and real-time exchanges mediated by technology (e.g., Skype, chat rooms) [5]. Studies relevant to pediatric mental health were also excluded as they are being evaluated separately in a systematic review currently underway. Outcomes were not defined *a priori* as they were to be incorporated into our description of the field.

### Data extraction and quality assessment

Data were extracted using standardized forms and entered into Microsoft Excel (Microsoft, Redmond, Washington, USA) by one reviewer and verified for accuracy and completeness by another. Reviewers resolved discrepancies through consensus. Extracted data included study and population characteristics, description of the social media tools used, objective of the tools, outcomes measured and results, and authors’ conclusions [7]. Studies that examined social media as one component of a complex intervention were noted as such.

Two reviewers independently assessed the methodological quality of included analytic studies and resolved disagreements through discussion. We used the Cochrane Risk of Bias tool to assess randomized controlled trials (RCTs) and non-randomized controlled trials (NRCTs) [8]. We used a tool for before-after studies that was developed based on the Newcastle-Ottawa Scale [9] and used in a previous review [10]. Quality assessment was not conducted on cross-sectional or qualitative studies as they were used to provide additional context to how social media is being used in child health, rather than to provide estimates of effect.

### Data synthesis and analysis

We described the results of studies qualitatively and in evidence tables. Descriptive statistics were calculated using StataIC 11 (StataCorp, College Station, Texas, USA). Studies were grouped according to target user and study design. When studies provided sufficient data, we calculated standardized mean differences and 95% CIs for the primary outcomes and reported all results in forest plots created using Review Manager, Version 5.2 (The Cochrane Collaboration, Copenhagen, Denmark). We did not pool the results as the primary outcome varied across studies; however, we displayed the information graphically to examine the magnitude of effect of the social media interventions.

## Results

**Figure 1:**
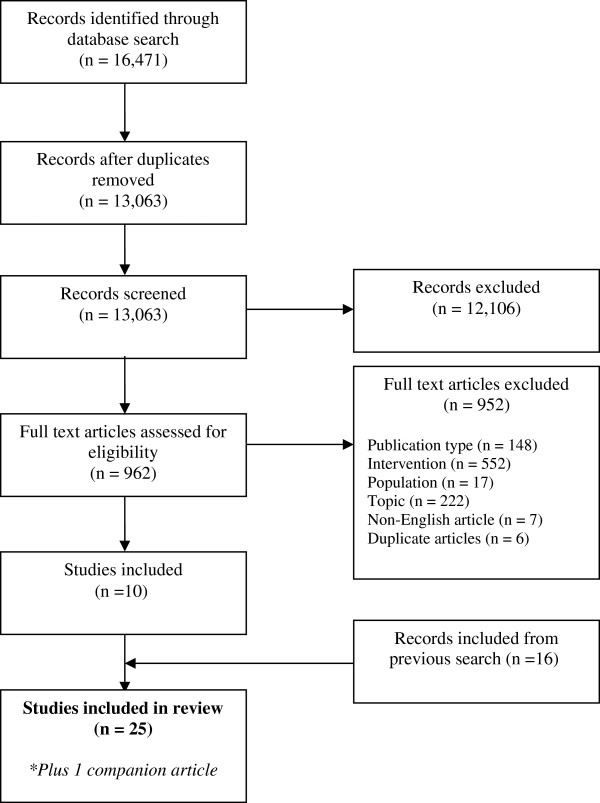
Flow diagram of study selection.

We identified 25 studies in 26 articles that met our inclusion criteria: 16 from the original search [5] and 10 from the update. Figure 1 outlines the flow of studies through the inclusion process and Table 1 provides a description of the included studies. The most common uses for social media were for health promotion (52%) and the tools were largely community-based (64%). Adolescents were more often the target audience (64%) than children (36%) or caregivers or families (44%; 40% targeted multiple groups). Discussion forums were the most commonly used tools (68%). Nearly all authors concluded that the social media tool evaluated showed evidence of utility (80%) and the remainder were neutral (20%); none reported negative conclusions.

**Table 1:** Description of included studies

**Variable**	**Total – n (%)**
***Total – N***	***25***
**Country of corresponding author**	
Australia	1 (4)
Canada	4 (16)
China	1 (4)
Sweden	1 (4)
Taiwan	1 (4)
USA	17 (68)
Study start date – median (range)	2007 (2000 – 2011)
Study duration – median (range)	7.5 months (1 – 45)
Sample size – median (range)	51 (12 – 1349)
**Publication type**	
Journal article	23 (92)
Dissertation	2 (8)
**Study design**	
*Quantitative*	*18 (72)*
Randomized controlled trial	8 (32)
Non-randomized controlled trial	2 (8)
Before-after	2 (8)
Cross-sectional	6 (24)
*Qualitative*	*5 (20)*
Ethnography	2 (8)
Content analysis	3 (12)
*Mixed methods*	*2 (8)*
**Study population**	
Children (<13 years old)	9 (36)
Youth (13–18 years old)	16 (64)
Caregivers	11 (44)
**Condition type**	
Acute	2 (8)
Chronic	10 (40)
Health Promotion	13 (52)
**Study setting**	
Hospital	6 (24)
Outpatients	3 (12)
Community	16 (64)
**Social media tool**	
Blog	1 (4)
Twitter	1 (4)
YouTube	1 (4)
Facebook	4 (16)
Social networking site	1 (4)
Virtual world (Zora)	2 (8)
Discussion forum	17 (68)
Component of a complex intervention	16 (64)
**Authors’ conclusions**	
Positive	20 (80)
Neutral	5 (20)
Negative	-

### How social media is being used in child health

While social media interventions were used to target health outcomes in children, youth, and caregivers, adolescents were the most commonly studied population. Two studies were based on acute conditions; 10 on chronic conditions, with clusters in type 1 diabetes (n = 3) and cancer (n = 2); and 13 for health promotion purposes, focusing mainly on healthy diet and exercise (n = 5), sexual health (n = 4), and smoking cessation (n = 2; Tables 2 and 3).

**Table 2:** Characteristics of included studies

**Author, Year (Country)**	**Study design, target population, and quality assessment**	**Objectives (Length of intervention)**	**Social media intervention**	**Comparator**	**Primary outcome measure (Tool)**
*Acute*					
Braner, 2004 (USA) [11]	Cross-sectional.	To describe an experience with a Web-based communications program for the patients, families, and referring physicians of patients admitted to the PICU. (Access to website during PICU stay)	Social networking site in which PICU nurses and family posted notes and messages, respectively. (n = 78)	NA	Satisfaction (survey).
	PICU patients, their families, and referring physicians.				
	NA				
Lim Fat, 2011 (Canada) [12]	Content analysis.	To assess YouTube videos on infantile spasms for quality and efficacy as an educational tool for parents and medical staff. (NA)	YouTube (n = 54 videos)	NA	Technical quality (Medical Video Rating System, designed by authors).
	Parents of infants experiencing infantile spasms.				
	NA				
*Chronic*					
*Cancer*					
Ewing, 2009 (USA) [13]	Mixed methods.	To develop a Web-based resource for families of children newly diagnosed with cancer. (Access to website for 6 months)	Discussion forum as part of a multimedia website including information on coping strategies, ask-an-expert and FAQ sections, and additional resources. (n = 21 families)	NA	Website usage (usage statistics, Website Evaluation Instrument).
	Families with a child (8–17 years) diagnosed with cancer in the past 6 weeks.				
	NA				
Nicholas, 2012 (Canada) [14]	Mixed methods.	To evaluate the effectiveness of the online peer support network. (Access to forum for 3 months)	Discussion forum monitored by social worker. (n = 19)	NA	Paternal coping, social support and meaning of illness (Coping Health Inventory for Parents, Multi-Dimensional Support Scale, Meaning of Illness Questionnaire).
	Fathers of children (4–17 years) with brain tumors.				
	NA				
*Juvenile Idiopathic Arthritis*					
Stinson, 2010 (Canada) [15]	Content analysis.	To explore the usability of a self-management program for youth with JIA and their parents to refine the health portal prototype. (NR)	Discussion forum as part of 12 modules containing content, graphics, video clips, interactive components, and animations. (n = 19)	NA	Ease of use (qualitative usability testing approach with semi-structured interviews and observation by a trained observer).
	Adolescents (mean 15.7 ± 1.5 years) with JIA.				
	NA				
*Renal Disease*					
Bers, 2001 (USA) [16]	Ethnography.	To determine if Zora is safe and satisfying for children with end-stage renal disease on hemodialysis. (NR)	Zora, a virtual world in which avatars can build a virtual city, chat with each other in real-time or through message boards, create virtual places and characters and write interactive stories. (n = 12)	NA	Satisfaction (survey and interview).
	Children (7–21 years) receiving dialysis for end-stage renal disease.				
	NA				
*Transplant*					
Bers, 2009 (USA) [17]	Content analysis.	To facilitate peer network-building amongst same-age pediatric post-transplant patients. (8 months)	Zora, a virtual world in which avatars can build a virtual city, chat with each other in real-time or through message boards, create virtual places and characters and write interactive stories. (n = 22)	NA	Description of pilot study (home visits, interviews, notes from parents and medical staff, and analysis of the participant chat log).
	Post-transplant children (11–15 years).				
	NA				
*Type 1 Diabetes*					
Merkel, 2012 (USA) [18]	Before-after.	To determine parents’ self-reported self-efficacy scores related to diabetes care management pre- and post-implementation of a Web-based social support platform. (6 weeks)	Discussion threads with area/national community resources and links, and diabetes camp information. (n = 14)	NA	Parental self-efficacy (Self-Efficacy for Diabetes Scale-Parent Modified; Diabetes Empowerment Scale-Short Form-Parent Modified).
	Parents of a child diagnosed with type 1 diabetes.				
	7/13 points (BAQA)				
	Moderate quality				
Nordfeldt, 2010 (Sweden) [19]	Content analysis.	Explore patients' and parents' attitudes toward a local Web 2.0 portal tailored to young patients with type 1 diabetes and their parents. (Accessed between 2006–2008)	Portal containing blogs, discussion forums and specific diabetes-related information. (n = 24)	NA	Attitudes toward the functionality of the web portal (interviews).
	Parents and pediatric patients (11–18 years) treated by diabetes teams, and their practitioners.				
	NA				
Whittemore, 2010 (USA) [20]	RCT	To develop an Internet coping skills training program. (4–5 weeks)	Discussion forum moderated by a health professional, along with information sessions presented through graphic novel models, and profile creation. (n = 6)	Four weekly sessions on glucose control, nutrition, exercise and sick days, and new technology. (n = 6)	HbA1C (blood test,%).
	Adolescents (13–16 years) with type 1 diabetes.				
	Unclear RoB				
	Moderate quality				
*Other*					
Baum, 2004 (USA) [21]	Cross-sectional.	To determine how primary caregivers of a child with special health care needs rate and describe their reasons for participating in an Internet parent support group in terms of problem-focused and emotion-focused coping. (NR)	Discussion forum for a peer support group. (n = 114)	NA	How problem-focused and emotion-focused coping are associated with reasons for participating in Internet parent support groups (survey).
	Parents of children (mean: males 6.5 years, females 8.7 years) with special health care needs.				
	NA				
Nicholas, 2007 (Canada) [22]	Ethnography.	To examine perceptions and experiences of children who use an online pediatric support network. (NR)	Discussion forum as part of an interactive network with other features including information, entertaining activities, chat rooms and videoconferencing. (n = 21)	NA	Perceived outcomes following hospitalized children’s participation in a pediatric online support network (“long interview”, based on semi-structured approach).
	Hospitalized children and adolescents (4–17 years), their parents/caregivers, and healthcare professionals.				
	NA				
*Health Promotion*					
*Healthy Diet & Exercise*					
Cordeira, 2012 (USA) [23]	Before-after.	To determine if the Young Leaders for Healthy Change Fall 2011 program had a significant effect on nutrition and physical activity behaviors; and known determinants of behavior, including: knowledge, beliefs, attitudes (self-efficacy and social support) in the domains of nutrition, physical activity, and advocacy. (12 weeks)	Facebook page plus 12 online education-based lessons, 2 online training programs, 12 peer/family weekly challenges, and a community service project. (n = 238)	NA	Participation in 60 minutes of physical activity every 5–7 days of the week (2008 Physical Activity Guidelines for Americans survey).
	High school students in grades 9–12.				
	4/13 points (BAQA)				
	Weak quality				
DeBar, 2009 (USA) [24]	RCT.	To test the efficacy of a health plan-based lifestyle intervention to increase bone mineral density in adolescent girls.	Youth Talk discussion board, online scrapbook page, psycho-educational information, diet and exercise goal and achievement records, “I Need” and “Ask a Health Question” forums, all available through a web-based study site; group and individual meetings; attendance at a retreat; and coaching telephone calls. (n = 113)	Social activities with discussions focused on general health issues rather than bone health specifically; no personalized feedback about behavioral goal attainment. (n = 115)	Bone mineral density (Dual Energy X-ray Absorptiometry).
	Girls aged 14–16 years with a body mass index below the national average.				
	High RoB				
	Weak quality				
Lao, 2011 (USA) [25]	RCT.	To evaluate the impact and feasibility of the Individual Nutrition Health Plans, a nutrition and exercise pilot curriculum focused on improving beverage choice, physical activity, fruit and vegetable consumption, and fast food consumption behaviors. (8 weeks)	Individual Nutritional Health Plan administered through text, Facebook, and Twitter. (n = 106)	Wait list control. (n = 86)	Frequency of sweetened beverage consumption (survey).
	Hispanics or low-income high school students aged 14–17 years receiving health plans.				
	High RoB				
	Weak quality				
Rydell, 2005 (USA) [26]	RCT.	To promote bone mass gains among girls through increased intake of calcium-rich foods and weight-bearing physical activity. (2 years)	Discussion forum, girl scout troop meetings, home activities and summer camp. (n = 194)	Regular girl scout troop meetings. (NR)	Change in bone mineral content (Dual Energy X-ray Absorptiometry).
	Preadolescent girls aged 10–12 years.				
	High RoB				
	Weak quality				
Savige, 2005 (Australia) [27]	Cross-sectional.	To examine how one model of e-learning can be used to support the food and nutrition education of future learners. (NR)	Discussion forum along with information about food and nutrition, quizzes, story writing, interactive food activities, positive role model profiles, and games with food and nutrition themes. (n = 1349)	NA	Self-reported intake of food and drink (online survey including a 24-hour recall checklist).
	Primary school students in grade 4 and associated composite grades.				
	NA				
*Sexual Health*					
Cox, 2009 (USA) [28]	RCT.	To describe the development of a Web-based program to help mothers talk to their children about sex (CASE), and to pilot test the feasibility and efficacy of CASE.	Free access to monitored discussion board postings and discussion, professional advice, and e-mail. (n = 20)	Same information as the mothers in the intervention group, in notebook form. (n = 20)	Self-efficacy (Self-Efficacy of Parents to Discuss Sexual Health Issues with their Adolescents Scale).
	Rural, low-income mothers of children in grades 5–10.				
	High RoB				
	Weak quality				
Jones, 2012 (USA) [29]	Cross-sectional.	To evaluate an evidence-based social-networking intervention aimed at reducing the incidence of chlamydia among youth. (NR)	Facebook page including educational information, and links to videos and resources. (n = 70)	NA	Intention to engage in risky sexual behavior (survey).
	Youth aged 15–24 years.				
	NA				
Lou, 2006 (China) [30]	NRCT.	To evaluate the effectiveness of the website in increasing adolescents' and young people's knowledge and in changing their attitudes and behaviors regarding sex. (10 months)	Website including a discussion forum, information, videos and expert mailbox. (n = 624)	No sex education. (n = 713)	Knowledge score (survey).
	Adolescents and unmarried youth in China.				
	High RoB				
	Weak quality				
Yager, 2012 (USA) [31]	Cross-sectional.	To develop and evaluate a Facebook site, Teen Sexual Health Information; and to empower sexually active adolescents who viewed the site with confidential information to help them remain sexually healthy. (NR)	Facebook page with videos, photographs, fact sheets about sexually transmitted infections, free and reduced-cost clinic locations for testing and treatment, and links to other online resources. (n = 39)	NA	Website evaluation (survey).
	Adolescents aged 13–20 years.				
	NA				
*Smoking Cessation*					
Chen, 2006 (Taiwan) [32,33]	NRCT.	To develop an Internet-assisted smoking cessation program accompanied with auricular acupressure, and compare the quit rate and self-efficacy of youth smokers receiving auricular acupressure with and without the Internet-assisted smoking cessation program. (4 weeks)	Website with eight components: impact of smoking, auricular acupuncture for smoking cessation, critical issues in smoking cessation, online questionnaire, professional counseling, discussion forum, hot topics, and hyperlinked websites. (n = 38)	Auricular acupressure only. (n = 39)	Mean serum cotinine levels (cotinine direct ELISA kit and reader).
	High school seniors who smoke.				
	High RoB				
	Weak quality				
Patten, 2006 (USA) [34]	RCT.	To evaluate a novel treatment delivery method for smoking cessation. (24 weeks)	Discussion forum along with a gallery to post artwork, information services, videos of personal stories, private journaling, quizzes, quit plan and quit notes. (n = 70)	Four brief sessions with research counselors and homework assignments. (n = 69)	Point-prevalence smoking abstinence (Cigarette Timeline Followback interview, verified by expired breath carbon monoxide levels ≤8 parts per million).
	Adolescent smokers aged 11–18 years				
	High RoB				
	Weak quality				
*Other*					
Baggett, 2010 (USA) [35]	RCT.	To determine if parents would engage in an Internet-delivered intervention to support their infant’s social-emotional development. (NR)	Information sharing via a discussion board, with multimedia presentation of concepts, behaviours, and skills; check-in questions; summary of key concepts; daily homework; video of mother-infant interactions for review by coach and parent; and a weekly telephone call from a coach to review content and provide personalized support. (n = 20)	Provision of computer and Internet connection, with links to infant development and parenting resources on the Internet. (n = 20)	Mother-infant interaction (Landry Parent–child Interaction Scales, free-play observation).
	Mothers of infants (3–8 months) at risk for poor social-emotional outcomes.				
	Unclear RoB				
	Moderate quality				
Hudson, 2012 (USA) [36]	RCT.	To test the effects of the New Mothers Network on single, low-income, adolescent, African American mothers’ psychological, parenting, and health care utilization outcomes. (6 months)	Discussion forum involving research nurse and peers, along with online educational information and e-mail access. (n = 21)	Usual care. (n = 21)	Depressive symptoms (20-item Center for Epidemiologic Studies Depression Scale).
	Single, low-income, adolescent (16–22 years), African American new mothers.				
	High RoB				
	Weak quality				

BAQA: Before-After Quality Assessment Tool; ELISA: enzyme-linked immunosorbent assay; FAQ: frequently asked questions; HbA1C: hemoglobin A1C; JIA: juvenile idiopathic arthritis; NA: not applicable; NR: not reported; NRCT: non-randomized controlled trial; PICU: pediatric intensive care unit; RCT: randomized controlled trial; RoB: Risk of Bias.

**Table 3:** Results for primary outcomes and conclusions of included studies

**Author, Year (Study design)**	**Authors’ Conclusions**	Statistically Significant*	**Conclusions**
*Acute*			
Braner, 2004 [11] (Cross-sectional)	Positive	NA	Families and referring physicians found the web-based communications to be helpful during a child’s pediatric intensive care unit hospitalization.
Lim Fat, 2011 [12] (Content analysis)	Positive	NA	YouTube may be an efficient teaching tool for infantile spasms, based on the number and quality of videos available. Education regarding effective search and selection practices is important to take advantage of YouTube as an information resource.
*Chronic*			
*Cancer*			
Ewing, 2009 [13] (Mixed methods)	Neutral	NA	Usage of the website was lower in this study than what has been reported in similar populations. The timing and method by which families are introduced to the website may influence their future use of the site.
Nicholas, 2012 [14] (Mixed methods)	Positive	Yes	Fathers of children with a brain tumor are an underserved clinical population at considerable emotional risk. Online social support resources may facilitate paternal coping.
*Juvenile Idiopathic Arthritis*			
Stinson, 2010 [15] (Content analysis)	Positive	NA	Support for the usability of the Teens Taking Charge: Managing Arthritis Online treatment program for youth with juvenile idiopathic arthritis appears to be strong. Online self-management programs for youth with chronic health conditions increase the accessibility and acceptability of treatments to youth unable to obtain these services in their local communities.
*Renal Disease*			
Bers, 2001 [16] (Ethnography)	Positive	NA	Through Zora, dialysis patients were able to express themselves and explore aspects of their identity that are usually underplayed during treatment. Patients had the ability to privately interact with others in similar situations, share opinions about their medical treatment and contribute to social support networks.
*Transplant*			
Bers, 2009 [17] (Content analysis)	Positive	NA	Zora was well-received by patients, parents and medical staff. The program brought about general satisfaction and changes in some patients.
*Type 1 Diabetes*			
Merkel, 2012 [18] (Before-after)	Positive	Yes	Online social support is a feasible, cost-effective and low maintenance approach to healthcare management. Participants noted that the safe and secure environment for sharing life experiences related to the care of a child with type 1 diabetes was a major benefit of the online support group.
Nordfeldt, 2010 [19] (Content analysis)	Positive	NA	Web 2.0 services may help parents and patients with type 1 diabetes retrieve information and manage their condition. Health care professionals should be committed to maintaining and updating this information to support continued use of online resources.
Whittemore, 2010 [20] (RCT)	Positive	No	The group-based computer skills training intervention, TEENCOPE, was feasible and acceptable for adolescents with type 1 diabetes. Preliminary findings suggest that TEENCOPE improves select health outcomes in this population and indicate effect sizes for a future clinical trial.
*Other*			
Baum, 2004 [21] (Cross-sectional)	Positive	NA	Internet Parent Support Group (IPSG) may be a valuable resource to help parents understand and manage their children with special health care needs, especially for mothers under the stress of dealing with a chronically ill child. This study found that IPSG participation benefited caregiver-child relationships and ability to relax, but not health habits.
Nicholas, 2007 [22] (Ethnography)	Positive	NA	Online networks are promising resources for children and tools for promoting family-centered care. Online interventions contribute to enhanced self-esteem, reduced depression and other important child health outcomes, and appear to be promising as an augmenting source of psychosocial support.
*Health Promotion*			
*Healthy Diet & Exercise*			
Cordeira, 2012 [23] (Before-after)	Neutral	No	Programs promoting healthy behaviors for high school students require different strategies than maintaining healthy behaviors. Tailoring a program to meet the needs of all students may increase the potential reach of the program.
DeBar, 2009 [24] (RCT)	Positive	No	This health care-based lifestyle intervention demonstrated significant increases in bone mineral density in the spine and femoral trochanter of girls aged 14–16 with low body mass index. Furthermore, the intervention increased dietary calcium, vitamin D, and fruit and vegetable consumption during a 2-year period.
Lao, 2011 [25] (RCT)	Neutral	No	Social media has the ability to reach a large population with minimal effort; however, engaging at-risk students in health-promoting web groups and news feeds is difficult.
Rydell, 2005 [26] (RCT)	Neutral	No	This community-based behavioral intervention aimed at increasing dietary calcium intake and weight bearing physical activity (WBPA) was not effective in increasing bone mass gains, or frequency of WBPA, over a two-year period.
Savige, 2005 [27] (Cross-sectional)	Positive	NA	The website was successful in promoting children's interest in food. Nutrition promotion websites are beneficial because children are allowed to interact with their peers beyond the classroom, and websites can accommodate the changing needs of children.
*Sexual Health*			
Cox, 2009 [28] (RCT)	Positive	No	The web-based intervention was equally effective at improving mothers’ knowledge, communication skills, and self-efficacy as the written material control. Low-income, rural women with little to no prior computer experience can effectively learn and communicate online health information to their adolescents.
Jones, 2012 [29] (Cross-sectional)	Positive	NA	This study provides preliminary support for the use of social media, particularly Facebook, as an information dissemination and positive behavioral change tool for 15- to 24-year-olds.
Lou, 2006 [30] (NRCT)	Positive	Yes	Internet education programs increased students' health knowledge of reproduction and had some influence on attitudes, but no influence on behavior.
Yager, 2012 [31] (Cross-sectional)	Positive	NA	Using Facebook to circulate information to the adolescent population is a viable option. Full awareness of the potential risks and benefits of using social networking is important before dissemination of information via social media becomes common practice.
*Smoking Cessation*			
Chen, 2006 [32,33] (NRCT)	Positive	NR	The auricular acupressure and internet-assistant smoking cessation program significantly improved quitting rate and self-efficacy of the participants. Auricular acupressure can be safely self-administered by adolescents under the guidance of health educators. Adolescents may feel self-control and self-regulation through this method, and be more confident in facing the challenges of smoking cessation.
Patten, 2006 [34] (RCT)	Neutral	No	This home-based internet-delivered intervention was ineffective for adolescent smoking cessation. Abstinence rates were higher in the control group (not significant); however, intervention participants showed greater progress and reduction in the number of days smoked.
*Other*			
Baggett, 2010 [35] (RCT)	Positive	No	An internet-based intervention aimed at promoting infant social-emotional behavior through sensitive, responsive interactions with mothers may be beneficial. Online programs can reach families and professionals who may not otherwise be able to access direct intervention services or individualized training and ongoing support.
Hudson, 2012 [36] (RCT)	Positive	No	The New Mothers Network website may be an effective intervention for providing social support, especially emotional/appraisal support, to single, low-income adolescent mothers. Self-esteem was significantly higher in the intervention group.

*Statistical significance of primary outcome, if applicable.

NA: not applicable; NR: not reported.

### Acute conditions

Two studies evaluated social media as an intervention in an acute context: one in families of patients in the pediatric intensive care unit [11] (PICU) and one to help parents of children experiencing infantile spasms [12]. While both focused on pediatric health conditions, social media use was directed towards knowledge translation efforts, providing a source of information for the caregivers. The authors concluded that the interventions were beneficial to parents in both cases (Table 3).

### Chronic conditions

Two studies conducted in populations with chronic health conditions evaluated Zora, a virtual world [16,17], and the remaining eight studies investigated the use of discussion forums (Table 2) [13-15,18-22]. In each of the 10 studies, the primary objective of the social media tool was to provide support to the pediatric patient (n = 3), the parent or family of the patient (n = 3), or both (n = 4). These tools were often in the form of multi-faceted interventions (n = 5), in which the discussion forum was just one component. Discussion forums appeared alongside condition-specific information, interactive features, and links to other online and physical resources such as camps and support groups. Conditions included cancer, juvenile idiopathic arthritis, renal disease, organ transplants, and type 1 diabetes. Adolescents were included in all studies that were intended for the patient; children, ranging from 4 to 12 years old, were also included in five of these studies [13,16,17,19,22]. Outcomes in this group of studies were nearly all related to coping or self-efficacy, or perceptions and usage of the intervention. One study measured the impact of the social media tool on a change in a health outcome [15].

### Health promotion

Thirteen studies evaluated the use of social media as a health promotion tool. Health promotion efforts were directed at children and adolescents, but rarely at caregivers. The two studies that were exclusively aimed at children were focused on healthy diet and exercise, made use of discussion forums, and were aimed towards children from 8 to 12 years old [26,27]. The two studies evaluating tools intended for caregivers used online educational strategies incorporating discussion forums [28,35]. The remaining nine studies evaluated social media use in adolescent populations, covering healthy diet and exercise [23-25], sexual health [29-31], smoking cessation [32,34], and parenting issues [36].

The largest proportion of health promotion studies was in the area of healthy diet and exercise [23-27]; three of these five studies have been examined in more detail in another systematic review conducted by our group [37]. Although most of the social media tools evaluated were discussion forums, there was more variety in the health promotion studies in the interventions used than for acute or chronic conditions, including investigations of existing, widely used platforms such as Facebook. Four studies evaluated the use of Facebook as an outreach strategy to engage with adolescents in a school setting or in the general public [23,25,29,31]. The primary goals of the interventions in the health promotion category were to effect change in health outcomes, or to act as educational resources.

### Effectiveness of social media in child health

We included 12 studies that evaluated the effectiveness of a social media intervention versus a comparator; their results are described below.

### Randomized controlled trials

Data were available from seven RCTs. Six trials evaluated discussion forums and all seven trials included the social media tool as a component of a complex intervention. Only one study [24] compared two social media interventions; two compared the social media intervention to an online tool without the social media component [20,35], two used a non-technological aspect as the comparison group [34,36], and two compared social media to no intervention [25,26]. In all seven RCTs, there was no significant difference in the primary outcome measured between the social media group and the comparator. Two studies [24,36] used composite measures as primary outcomes and significant benefits were found in individual components. The statistical significance of the primary outcomes in these seven RCTs is in contrast to the authors’ conclusions, in which four studies concluded that the social media intervention had a positive effect (Table 3).

Quality assessment of the RCTs was conducted using the Risk of Bias tool (Table 2) [8]. Two trials were at unclear risk of bias [20,35] and the remaining five were at high risk of bias [24-26,34,36]. Sequence generation and allocation concealment were poorly reported. While the nature of the intervention precluded blinding of participants, blinding of outcome assessors was only reported in two studies [24,26]. Attrition was high, leading to high risk of bias in four studies [25,26,34,36]. Selective outcome reporting was not present in any of the studies.

### Non-randomized and before-after studies

One NRCT [32] and two before-after studies [18,23] included sufficient data to calculate standardized mean differences. Two studies found no significant difference between groups [23,32], and the other found a significant benefit of social media using one scale but not another [18]. Despite these results, two studies [18,32] claimed that the social media intervention had a positive effect (Table 3).

Using the Risk of Bias tool [8] for the NRCT, and the before-after quality assessment tool, all three studies were poor to moderate quality (Table 2). The before-after studies described the intervention well, but lacked detail on the representativeness of the samples and the reliability of the outcome measures. The non-randomized controlled trial was at high or unclear risk of bias regarding allocation, blinding, and incomplete outcome reporting.

### Cross-sectional studies

Two of the included cross-sectional studies evaluated health outcomes following exposure to a social media intervention [27,29]. One further cross-sectional study [12] found that approximately 60% of YouTube videos on infantile spasms screened depicted accurate portrayals and 18.5% of videos were considered to be excellent patient resources.

### Attributes of social media associated with effectiveness or lack of effectiveness

While the social media tools and the conditions and outcomes they aim to address vary widely, we used descriptive, qualitative, and mixed methods studies to explore whether there were overlapping characteristics of social media generally that may help to explain their effectiveness or lack thereof.

### Cross-sectional studies

Three cross-sectional studies were used to evaluate users’ experiences with social media interventions. Yager et al. [31] investigated the use of a Facebook site as a source of information on sexual health, and found that adolescents appreciated the availability of this resource and the comprehensiveness of the information. Braner et al. [11] and Baum et al. [21] both evaluated tools used to provide support to parents and caregivers and found that user satisfaction was driven by finding the right balance between providing informational and emotional support.

### Qualitative and mixed methods studies

All seven of the qualitative and mixed methods studies that met our inclusion criteria evaluated social media tools that were intended to provide support to their users. Two studied a virtual world and five studied discussion forums.

The two evaluations of Zora, a virtual world designed to help young users explore identity issues, highlighted different aspects of what made the tool successful. In children and adolescents with end-stage renal failure undergoing dialysis, Zora provided an opportunity for distraction from their illness, and a means by which to escape their reality [16]. While healthcare providers felt that it would be an ideal tool to teach their patients about their illness, none of the children endorsed this view. Conversely, in adolescents who had undergone an organ transplant, communication with others who had been through the same experience built a sense of community and was a method of coping with their conditions [17].

Mirroring the findings related to the virtual world, the studies evaluating discussion forums found a dichotomy in that some groups preferred to use the social media tools as a distraction from their illness [13,22], while others valued the ability to connect with others [14,15,19]. One distinction between these two groups was that younger children tended to be included in those studies that presented social media as a diversion, while adolescents were more highly represented in studies that found a benefit in building a social network. In one study of fathers of a child with a brain tumor, connections with others helped them cope by limiting isolation and normalizing their experiences [14]. However, other studies found that parents and caregivers were less likely to feel comfortable participating in an online forum [13,15]. Other features identified as being important included maintaining a certain level of activity on the forum in order to attract returning users [19], and minimizing access barriers related to technology, such as the use of passwords [19,22].

## Discussion

The use of new technology such as social media in child health is rapidly expanding and evolving. This systematic review provides a comprehensive review of the research that has been conducted in order to serve as a resource to guide future clinical and research efforts in this area.

The majority of studies included in our review investigated the use of a discussion forum and assessed social media as one component of a complex intervention. Discussion forums were often intended to provide support, and were particularly prevalent in studies of chronic conditions. While there were reported benefits in this context, especially from the qualitative investigations, none of the studies that used a discussion forum to effect change in a health outcome reported significant results. Given the focus on health promotion in this group of studies, and the familiarity that children and youth have with current social media platforms, outreach strategies may be more effective if efforts are made up-front to identify the tools that the target audience is already using and tailor the intervention accordingly, rather than expect the group to find and adopt new technologies that have been developed to suit a more limited, condition-specific purpose.

The use of complex interventions limits the ability to tease out any effects specific to the social media component; however, the inclusion of qualitative research helped to provide context to some of the attributes that were perceived to be effective. In general, users were most drawn to the ability of social media to facilitate the development of a support network. This did not apply to all populations, though, and tended to be more relevant to older children, and parents or caregivers. In the study populations including younger children, the reported advantage of the social media intervention tended to lie within its ability to serve as a distraction from their illness.

The use of social media varied according to the type of condition studied. Tools applied to acute conditions targeted the parents, and were intended to be educational resources and promoted information sharing. As described above, provision of support, either to the patient or to the caregiver, was the primary goal in chronic conditions. Health promotion was the category that most directly targeted children and youth, and demonstrated the greatest variety in tools used and outcomes measured, perhaps indicating potential for future development.

The authors’ conclusions tended to be positive with respect to the effectiveness and promise of social media as an intervention or promotional tool for child health. However, this was rarely supported by the statistical significance of the results. While this is a new technology with plenty of advocates and hypothesized benefits, there is no clear evidence that it is effective in improving health outcomes in children and youth. The included studies were of poor to moderate quality and many did not use rigorous study designs. Moving forward, an emphasis on well-conducted experimental designs and qualitative research, rather than on descriptive studies, would help to illuminate whether social media demonstrates effectiveness, and what characteristics contribute to that potential impact. This could include an increased focus on head-to-head comparisons of the effectiveness of specific social media tools, qualitative evaluations of their perceived strengths and limitations, and the use of changes in health outcomes as end-points.

### Strengths and limitations

We conducted a comprehensive systematic review of the literature following well-endorsed methods [38,39]. Our results provide a view of how social media is being used in child health, including the target audiences and intended purposes. We included all study designs including qualitative and descriptive research in order to provide information to help understand how these interventions may or may not be effective. These results may help inform the development of future interventions and research. The limitations of this review pertain to the nature of the topic and the methodological quality of the primary research. Specifically, in the majority of studies, social media was used as one component of a complex intervention making it difficult to tease out the specific impact of the social media modalities. Further, the primary research in this area had methodological limitations that should be addressed in future research. For example, in randomized and non-randomized trials, attention should be focused on adequate sequence generation, allocation concealment, and blinding of outcome assessment. Attrition was problematic and may be reflective of the challenge of maintaining engagement with the target audience through the interventions employed. Integrating end-user engagement in the development of the social media tools or strategy may be a step in overcoming this obstacle in future research [40]. Finally, while this review provides a thorough examination of the academic literature, the nature and evolution of social media has left a gap between tools that are currently used and those that have been scientifically evaluated. Other tools may have been developed that were not captured in our search of formal academic investigations. Therefore, the findings of this review may point towards an absence of evidence, rather than evidence of the absence of effectiveness of social media as a strategy for health-related interventions in children and youth.

## Conclusions

This systematic review demonstrates that social media is being used extensively in child health for a variety of purposes. The most common uses for social media were for health promotion with a focus on healthy diet and exercise, sexual health, smoking cessation, and parenting issues. Adolescents were the most common target audience, discussion forums were the most commonly used tools, and the tools were largely community-based. Nearly all studies concluded that the social media tool evaluated showed evidence of utility; however, results of the primary outcomes from the majority of comparative studies showed no significant effect. This synthesis offers an important resource for those who are developing and evaluating interventions involving social media.

## Abbreviations

NRCT: Non-randomized controlled trial; PICU: Pediatric intensive care unit; RCT: Randomized controlled trial.

## Competing interests

The authors declare that they have no competing interests.

## Authors’ contributions

MPH, SDS, and LH designed the study. MPH coordinated the project and is the guarantor. AM conducted the literature search and contributed to drafting the manuscript. MPH, JS, GW, and AM screened articles and performed data extraction and quality assessment. MPH and LH interpreted the data. MPH drafted and all authors critically reviewed the manuscript. All authors read and approved the manuscript.

## Pre-publication history

The pre-publication history for this paper can be accessed here:

http://www.biomedcentral.com/1471-2431/14/138/prepub

## Supplementary Material

Additional file 1 Sample Search Strategy (Medline). Click here for file
