# Supplementary material for: A systematic review of the use and effectiveness of social media in child health
Source: BMC Pediatr. 2014 Jun 2;14:138. doi: 10.1186/1471-2431-14-138 (PMC4047773; doi:10.1186/1471-2431-14-138)
Supplement: Additional file 1 — Sample Search Strategy (Medline). [file 1471-2431-14-138-S1.docx]

**Additional file. Sample Search Strategy (Medline)**

Database: Medline via Ovid <1946 to Present>

Search Title: Social Media All Conditions Update 1.0 | Medline – 24 April 2013 -- AM

Date Searched: 3 May 2013

Results: 2,007

| *Internet and social media related MeSH:*  1. Computer-Assisted Instruction/  2. Computers/td, ut  3. Electronic Mail/  4. Mass Media/td, ut  5. Medical Informatics/  6. Online Systems/td, ut  7. Search Engine/  8. User-Computer Interface/  9. exp Internet/ |
| --- |
| *Internet and social medial related keywords:*  10. blog*.mp.  11. e-health.mp.  12. Facebook*.mp.  13. (forum* adj3 (internet or web* or chat*)).mp.  14. Googl*.mp.  15. "Health 2.0".mp.  16. "Medicine 2.0".mp.  17. microblog*.mp.  18. myspace.mp.  19. (online or on-line).mp.  20. PatientsLikeMe.mp.  21. podcast*.mp.  22. Second Life.mp.  23. (social adj3 media*).mp.  24. (Social adj3 network*).mp.  25. (twitter or tweet*).mp.  26. user generated content.mp.  27. (virtual adj3 (world* or communit*)).mp.  28. ("Web 2.0" or "Web 2").mp.  29. web-based.mp.  30. WebMD.mp.  31. (website* or web site* or webpage* or web page*).mp.  32. wiki*.mp.  33. World Wide Web.mp.  34. YouTube.mp. |
| 35. or/1-34 [Internet/social media MeSH and keywords] (136,622) |
| *Health care education/promotion terms*  36. Communication/  37. "Delivery of Health Care"/  38. health behavior/  39. Health Communication/  40. Information Dissemination/  41. Information Seeking Behavior/  42. Information Services/  43. "Information Storage and Retrieval"/  44. Patient Care/  45. social support/  46. exp Attitude to Health/  47. exp Health Education/  48. exp Health Promotion/  49. exp Health/  50. exp Self Care/  51. exp Self-Help Groups/  52. (health adj3 (behavio?r* or care or communicat* or educat* or promot* or service*)).tw.  53. (inform* adj3 (disseminat* or retriev* or seek* or service*)).tw.  54. (self adj3 (care or help or support*)).tw. |
| 55. **or/36-54** [MeSH words for health promotion/information dissemination] (1,021,580) |
| 56. **and/35,55** [combination of social media + health information terms] (39,253) |

Search filters to stream out non-research papers

| *RCT Filter*  57. randomized controlled trial.pt.  58. controlled clinical trial.pt.  59. randomized.ab.  60. placebo.ab.  61. exp Clinical Trials as Topic/  62. randomly.ab.  63. trial.ti.  64. or/57-63  65. exp animals/ not humans.sh.  66. **64 not 65** [Cochrane RCT filter to max sensitivity and precision] (796,178) |
| --- |
| *SR Filter*  67. meta analysis.mp,pt.  68. review.pt.  69. search*.tw.  70. **or/67-69** [HIRU SR filter to balance sensitivity and specificity] (1,922,766) |
| *Observational Study Filter*  71. epidemiologic studies/  72. exp Case-Control Studies/  73. exp Cohort Studies/  74. case control.tw.  75. (cohort adj (study or studies)).tw.  76. cohort analy*.tw.  77. (follow up adj (study or studies)).tw.  78. (observational adj (study or studies)).tw.  79. longitudinal.tw.  80. retrospective.tw.  81. cross sectional.tw.  82. Cross-Sectional Studies/  83. **or/71-82** [SIGN observational study filter] (1,681,223) |
| *Qualitative Research Filter*  84. interview*.tw.  85. experience*.mp.  86. qualitative.tw.  87. **or/84-86** [HIRU qualitative study filter] (828,027) |
| 88. **or/66,70,83,87** [combination of all search filters] (4,523,457) |
| 89. **and/56,88** [combination of social media + health + SD] (17,847) |
| 90. limit 89 to yr="2012 -Current" (2,234)  91. remove duplicates from 90 (2,007) |
